# Supplementary material for: Mapping the association between mental health and people’s perceived and actual ability to practice hygiene-related behaviours in humanitarian and pandemic crises: A scoping review
Source: PLoS One. 2023 Dec 14;18(12):e0286494. doi: 10.1371/journal.pone.0286494 (PMC10721104; doi:10.1371/journal.pone.0286494)
Supplement: S1 Table — (DOCX) [file pone.0286494.s001.docx]

**S1 Table.**

| **Concept** | **Broad search terms** |
| --- | --- |
| Humanitarian Crisis | disaster OR flood OR landslide OR typhoon OR tsunami OR hurricane OR drought OR famine OR outbreak OR war OR conflict OR humanitarian crisi*s OR war zone OR migrants OR refugees OR guests OR displaced person* |
| Hand Hygiene | handwashing with soap OR handwashing OR hand cleaning with saniti*er OR hand disinfection OR hand cleaning |
| Mental Health | Post-traumatic stress disorder OR PTSD OR anxiety OR depression OR mental health OR emotional distress OR psychosocial support OR personal wellbeing OR trauma OR distress OR trauma OR distress |
| Low- and Middle-income Country | low income country OR LIC or low?income OR middle income country OR MIC OR middle?income OR LMIC OR developing country OR developing countries OR Afghanistan OR Burkina Faso OR Guinea-Bissau OR Somalia OR Democratic People’s Republic of Korea OR North Korea OR South Sudan OR Burundi OR Liberia OR Sudan OR Syria* OR Central African Republic OR Madagascar OR Chad OR Malawi OR Togo OR Democratic Republic of Congo OR Mali OR Uganda OR Eritrea OR Mozambique OR Republic of Yemen OR Ethiopia OR Niger OR Gambia OR Rwanda OR Guinea OR Sierra Leone OR Angola OR Honduras OR Philippines OR Algeria OR India OR Samoa OR Bangladesh OR Indonesia OR São Tomé and Principe OR Belize OR Islamic Republic of Iran OR Senegal OR Benin Or Kenya OR Solomon Islands OR Bhutan OR Kiribati Or Sri Lanka OR Bolivia OR Kyrgyzstan OR Tanzania OR Cabo Verde OR Lao OR Tajikistan OR Cambodia OR Lesotho OR Timor Leste OR Cameroon OR Mauritania OR Tunisia OR Comoros OR Federal States of Micronesia OR Ukraine OR Republic of Congo OR Mongolia OR Uzbekistan OR Côte d’Ivoire OR Morocco OR Vanuatu OR Djibouti OR Myanmar OR Vietnam OR Egypt OR Nepal OR West Bank OR Gaza OR Palestine OR El Salvador OR Nicaragua OR Zambia OR Eswatini OR Nigeria OR Swaziland OR Zimbabwe OR Ghana OR Pakistan OR Haiti OR Papua New Guinea OR Albania OR Gabon OR Namibia OR American Samoa OR Georgia OR North Macedonia OR Argentina OR Grenada OR Panama OR Armenia OR Guatemala OR Paraguay OR Azerbaijan OR Guyana OR Peru OR Belarus OR Iraq OR Romania OR Bosnia and Herzegovina OR Jamaica OR Russian Federation OR Botswana OR Jordan OR Serbia OR Brazil OR Kazakhstan OR South Africa OR Bulgaria OR Kosovo OR St Lucia OR China OR Lebanon OR St. Vincent and the Grenadines OR Colombia OR Libya OR Suriname OR Costa Rica OR Malaysia OR Thailand OR Cuba OR Maldives OR Tonga OR Dominica OR Marshall Islands OR Turkey OR Dominican Republic OR Mauritius OR Turkmenistan OR Equatorial Guinea OR Mexico OR Tuvalu OR Ecuador OR Moldova OR Fiji OR Montenegro |
